# Supplementary figures and images for: Reno-protective effect of IL-34 inhibition on cisplatin-induced nephrotoxicity in mice
Source: PLoS One. 2021 Jan 11;16(1):e0245340. doi: 10.1371/journal.pone.0245340 (PMC7799787; doi:10.1371/journal.pone.0245340)

A.

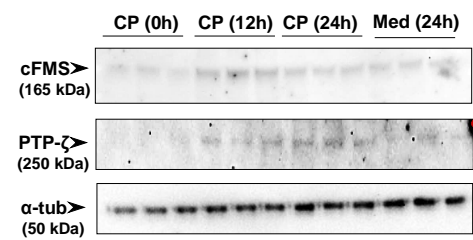

B.

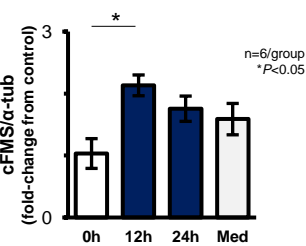

C.

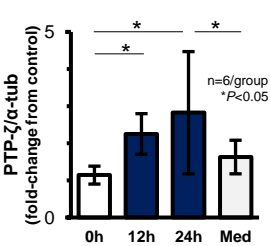

Supplement: S1 Fig — Representative WB analysis for cFMS, PTP-ζ, and α-Tub expression on MRPTEpiC after CP stimulation or the addition of medium (Med) for 0 to 24 h (A). Densitometric analysis of WB for cFMS (B) and PTP-ζ (C). The values shown are the values after normalization to α-Tub expression, and they are depicted as the relative ratio of cFMS or PTP-ζ to α-Tub. (PDF) [file pone.0245340.s002.pdf]

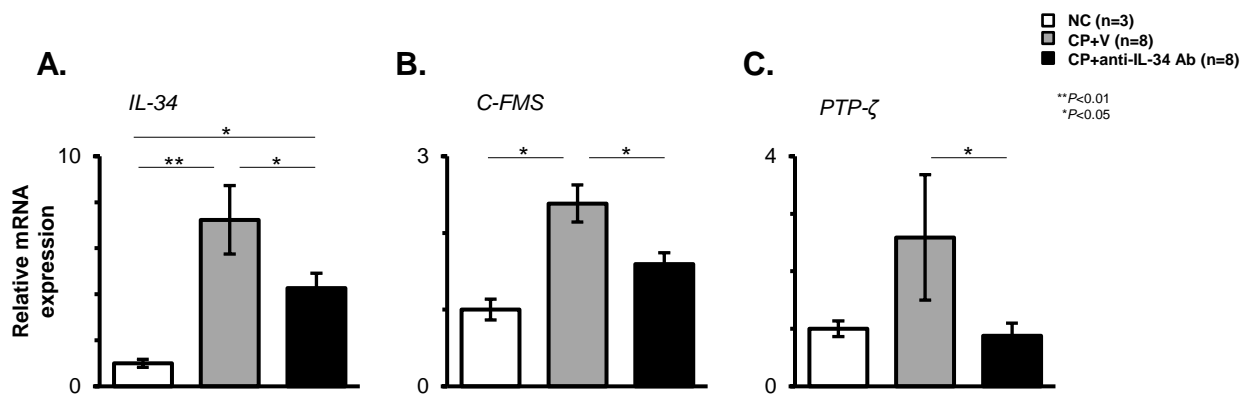

Supplement: S2 Fig — Real-time RT-PCR for IL-34 (A), C-FMS (B), and PTP-ζ (C) among the NC, CP+V, and CP+anti-IL-34 Ab groups of mice. Values were normalized to the GAPDH transcript, and are expressed as the relative ratio. Data are expressed as the mean ± SEM. The Mann-Whitney U test was used for statistical analysis. (PDF) [file pone.0245340.s003.pdf]

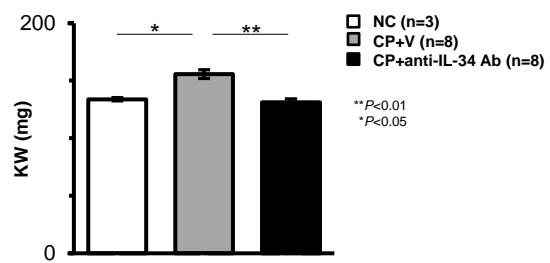

Supplement: S3 Fig — Kidney wight (KW) among the NC, CP+V, and CP+anti-IL-34 Ab groups of mice. Data are expressed as the mean ± SEM. The Mann-Whitney U test was used for statistical analysis. (PDF) [file pone.0245340.s004.pdf]

A.

Scheme

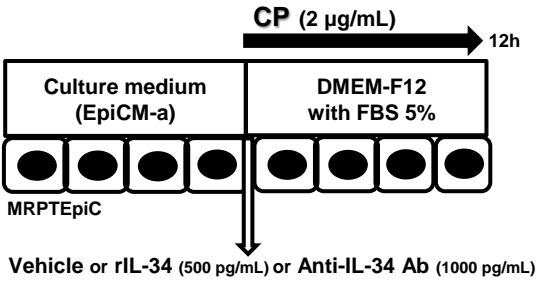

B.

Scheme

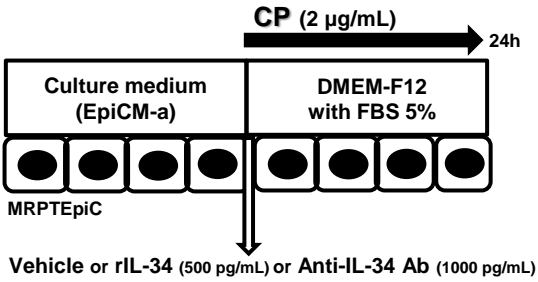

C.

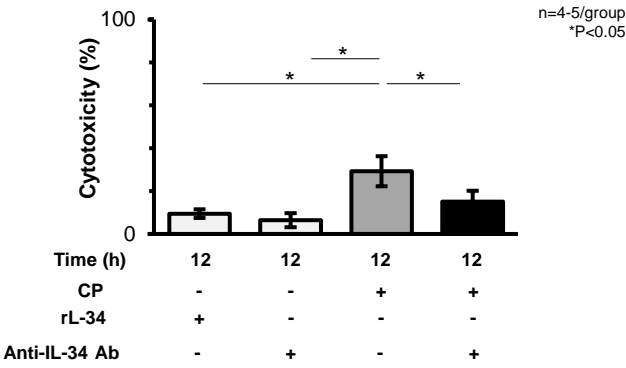

D.

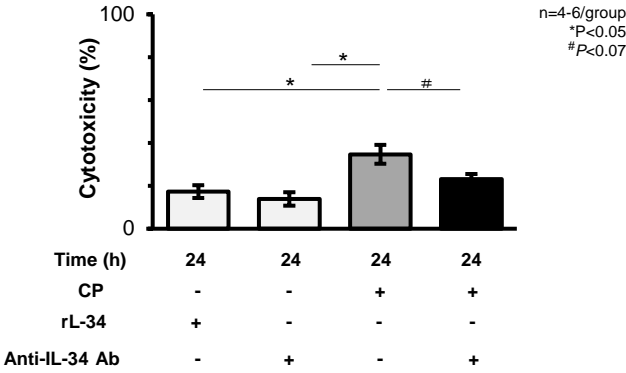

Supplement: S4 Fig — Cultured MRPTEpiC were stimulated with or without CP (2 μg/mL), followed by treatment with vehicle or rIL-34 (500 pg/mL) or anti-IL-34 Ab (1000 pg/mL) for 12 (A) or 24 h (B). Cytotoxicity in cultured TECs, evaluated by LDH assay, at the time of 12h (C) and 24h (D) were compared among the study groups. Values in the group without CP-stimulation and any treatment were recognized as control, and each value in the study groups was calculated using its control data. Data are expressed as the mean ± SEM. The Mann-Whitney U test was used for statistical analysis. (PDF) [file pone.0245340.s005.pdf]

***in vivo* (CP-N mice, Day 3)**

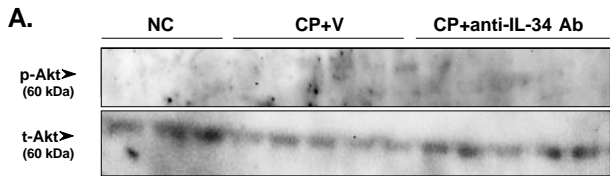

***in vitro* (MRPTEpiC, 6h)**

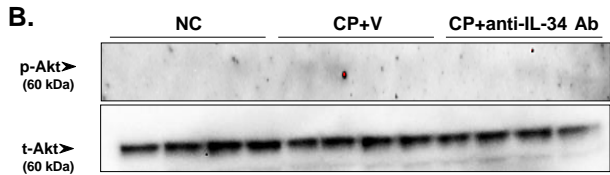

Supplement: S5 Fig — Representative WB analysis for p-Akt and t-Akt in renal cortical tissues after stimulation with CP for 72 h among the NC, CP+V, and CP+anti-IL-34 Ab groups of mice (A). Representative WB analysis for p-Akt and t-Akt in MRPTEpiC after stimulation with CP for 6 h among the NC, CP+V, and CP+anti-IL-34 Ab groups (B). (PDF) [file pone.0245340.s006.pdf]
