# Supplementary material for: Reno-protective effect of IL-34 inhibition on cisplatin-induced nephrotoxicity in mice
Source: PLoS One. 2021 Jan 11;16(1):e0245340. doi: 10.1371/journal.pone.0245340 (PMC7799787; doi:10.1371/journal.pone.0245340)
Supplement: S1 Table — (RTF) [file pone.0245340.s007.rtf]

S1 Table. Real-time RT-PCR primers to detect mRNAs.          
Gene                                                         Assay ID  
Act-â (â-actin)                                          �@�@�@ �@�@�@�@Mm02619580_g1
Bax                                                  �@�@�@�@�@Mm00432051_m1�@�@�@�@�@�@�@�@�@�@�@�@ 
Bcl-2                                                 �@�@�@�@ Mm00477631_m1
Csf-1                                                          Mm00432686_m1                 
Fer (cFMS)�@�@�@�@�@�@�@                                      �@�@�@Mm00484303_m1
Gapdh    �@�@�@�@�@�@�@                                       Mm99999915_g1                  
Il-1â                                                           Mm00434228_m1
Il-6                                                            Mm00446190_m1                    
Il-10                                                           Mm01288386_m1
Il-34                                              �@�@�@�@�@�@ Mm01243248_m1                 
Havcr1 (Kim-1)                                                 �@�@Mm00506686_m1
Mcp-1/Ccl2                                                     Mm00441242_m1                     
Mip1a/Ccl3                                                     Mm00441259_g1
Ptprz1                                                           Mm00478484_m1  �@�@�@�@�@�@�@�@�@�@�@�@�@�@�@�@�@�@�@�@�@�@�@�@�@�@�@�@�@�@�@�@�@�@�@�@�@�@�@�@�@�@�@�@�@�@�@�@�@�@�@�@�@�@�@�@�@�@�@�@�@�@�@�@�@�@  �@�@�@�@�@�@�@�@�@�@�@
Tnf-á                                                          �@�@Mm00443258_m1
