# Supplementary material for: Reno-protective effect of IL-34 inhibition on cisplatin-induced nephrotoxicity in mice
Source: PLoS One. 2021 Jan 11;16(1):e0245340. doi: 10.1371/journal.pone.0245340 (PMC7799787; doi:10.1371/journal.pone.0245340)
Supplement: S1 Raw image — (PDF) [file pone.0245340.s008.pdf]

a. Fig.3F

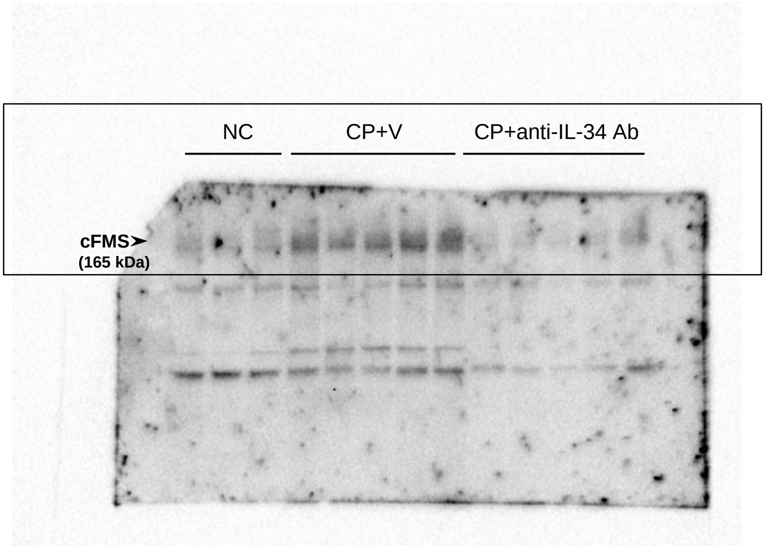

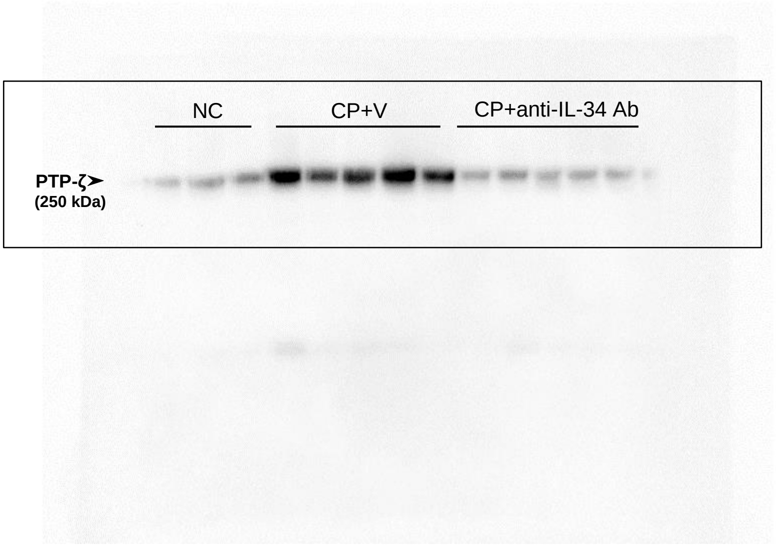

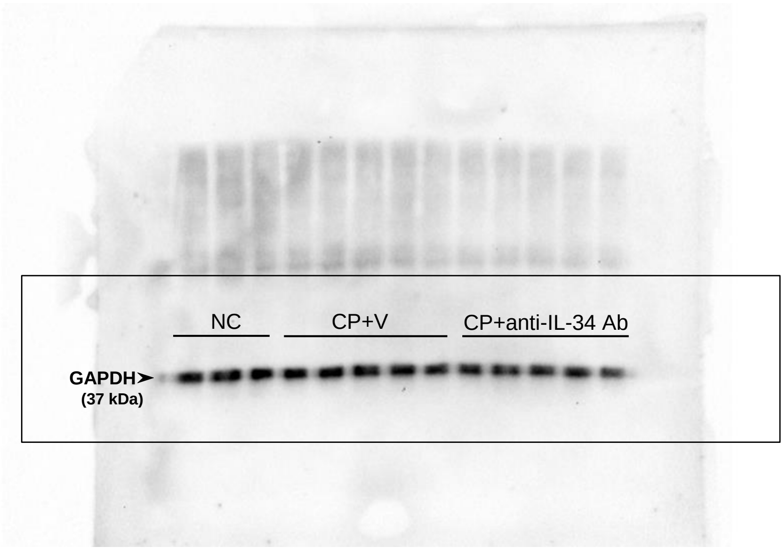

Captured by ChemiDoc MP Imaging System

b. Fig.8F

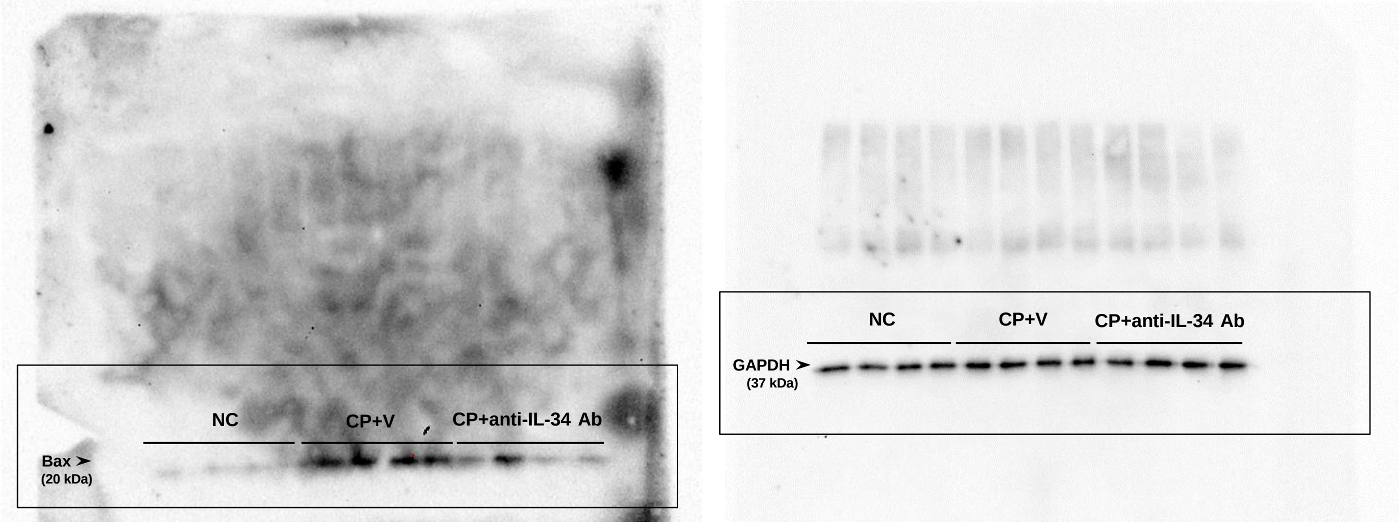

Captured by ChemiDoc MP Imaging System

c. Fig.10A and C

in vivo (CP-N mice, Day 3)

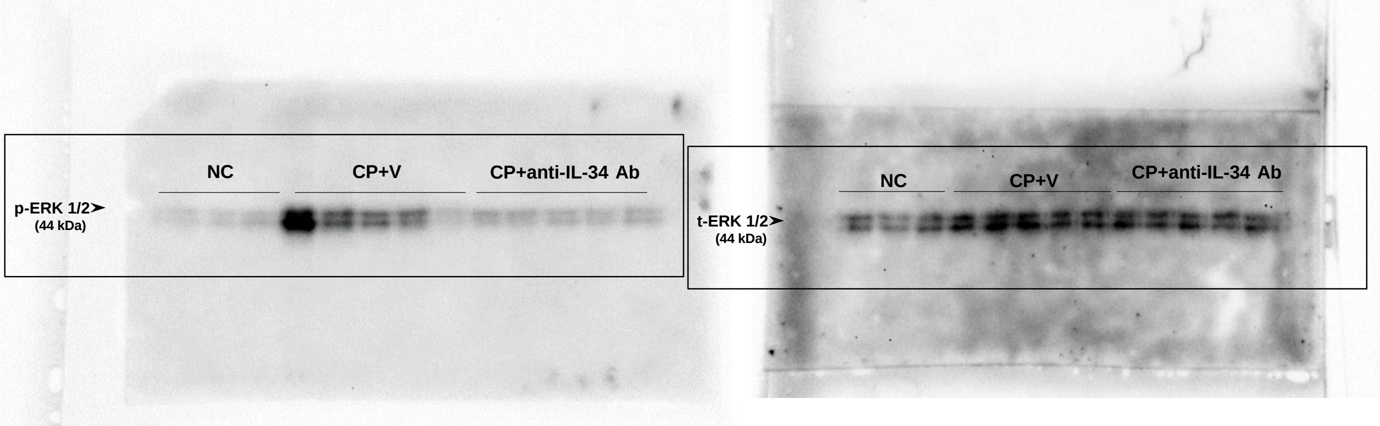

in vitro (MRPTEpiC, 6h)

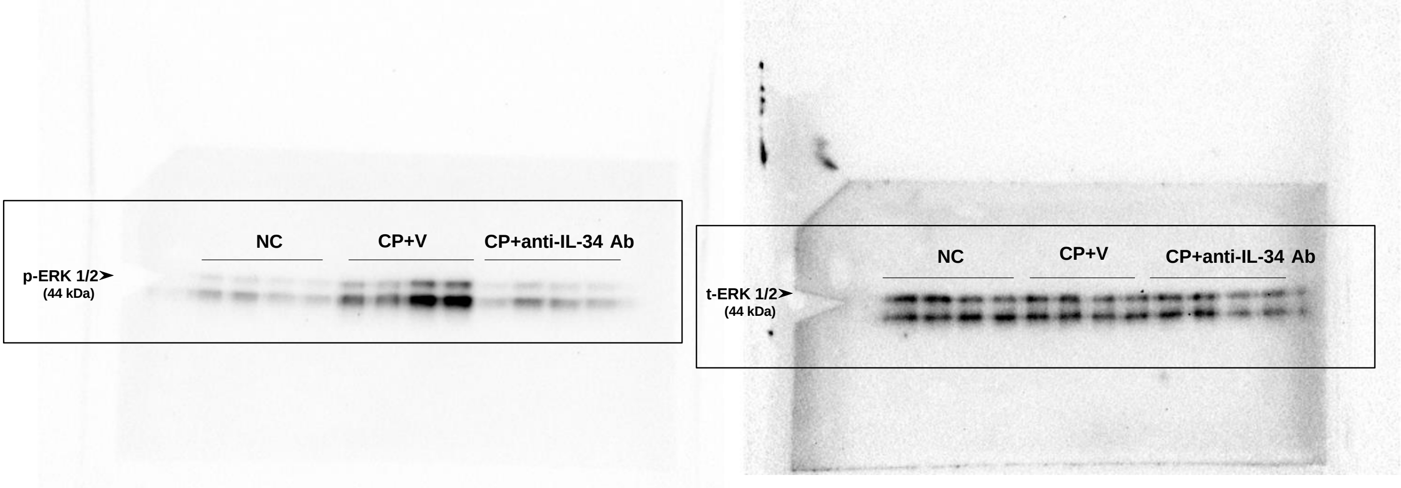

Captured by ChemiDoc MP Imaging System

d. S1Fig.1A

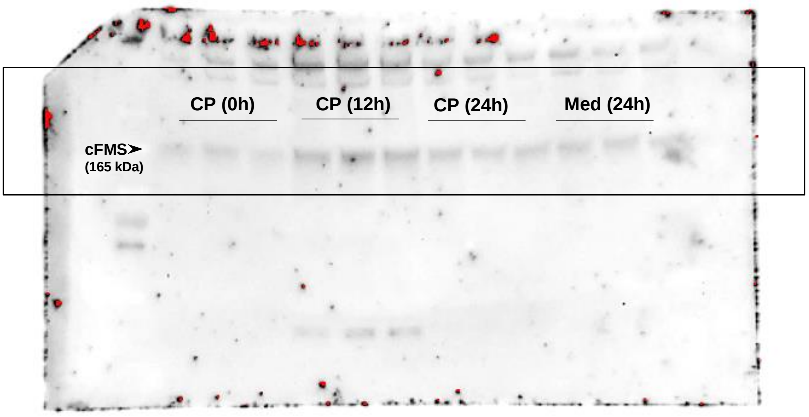

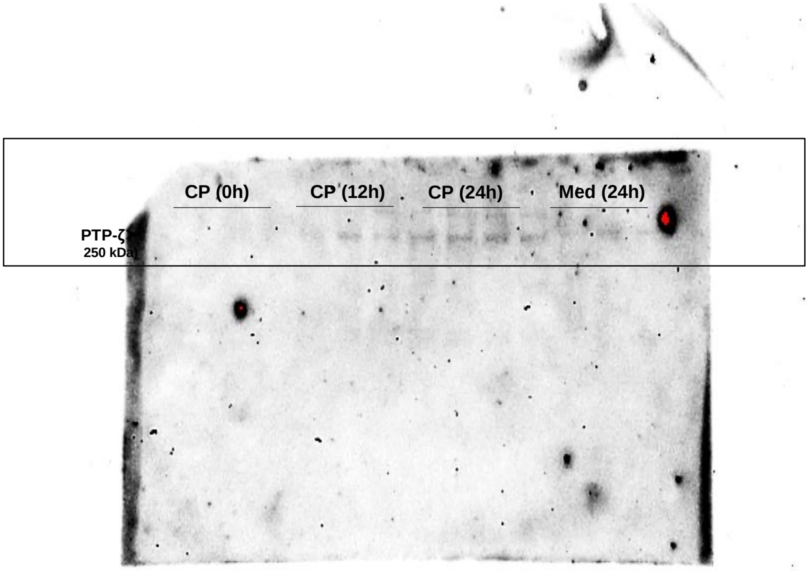

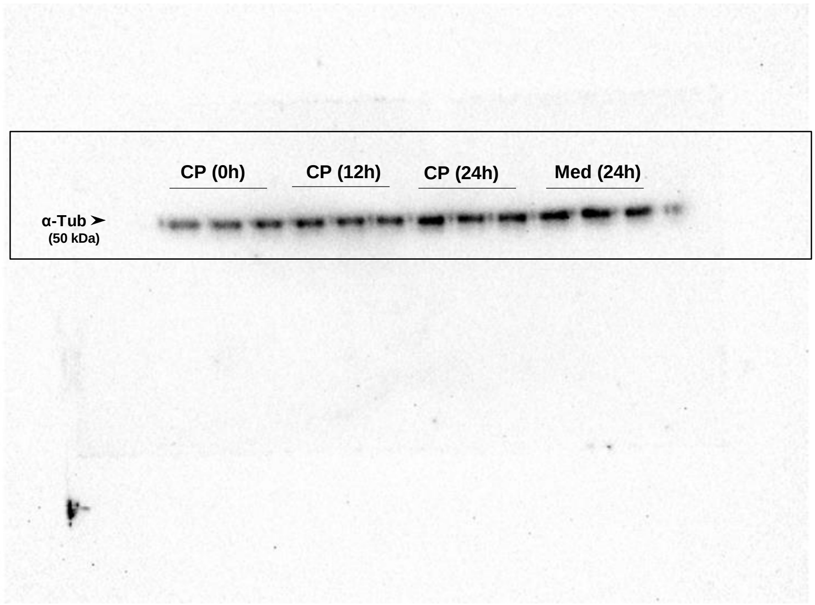

Captured by ChemiDoc MP Imaging System

e. S5Fig.A and B

***in vivo* (CP-N mice, Day 3)**

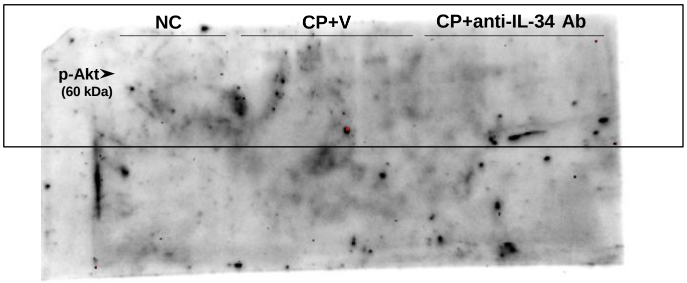

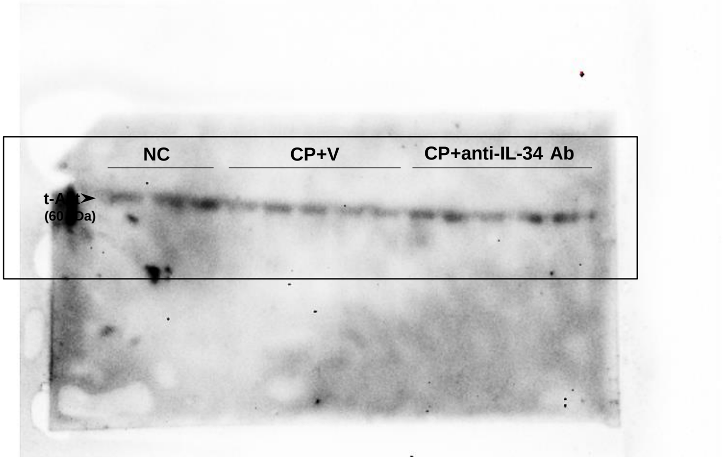

***in vitro* (MRPTEpiC, 6h)**

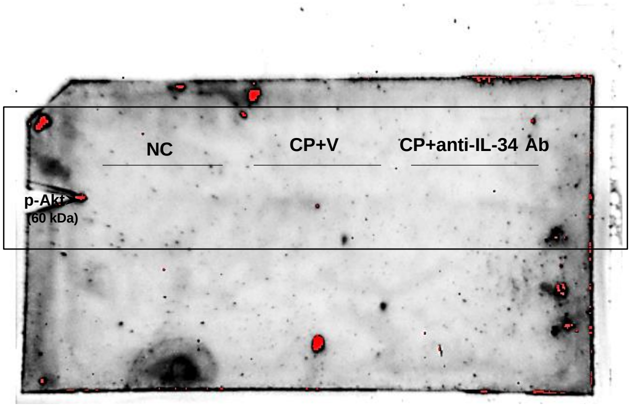

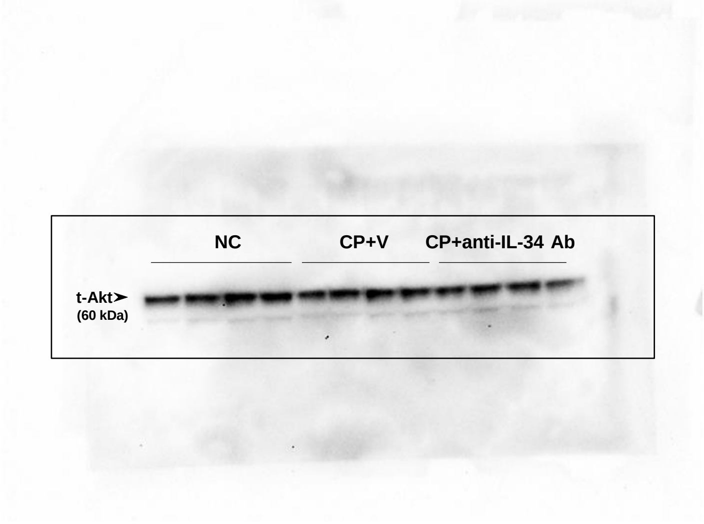

Captured by ChemiDoc MP Imaging System
